# New Hybrid Benzothiazole Derivatives from Gallic and Syringic Acid as a Potential Multifunctional Skin Disease

**DOI:** 10.3390/molecules31132245

**Published:** 2026-06-25

**Authors:** Leonardo Montani, Chiara Tupini, Filippo Marchetti, Alessandra Rizzo, Silvia Vertuani, Stefano Manfredini, Ilaria Lampronti, Anna Baldisserotto

**Affiliations:** 1 Department of Life Sciences and Biotechnology, Section of Medicine and Health Products, University of Ferrara, Via Fossato di Mortara 19, 44121 Ferrara, Italy; leonardo.montani@unife.it (L.M.); filippo.marchetti@unife.it (F.M.); vrs@unife.it (S.V.); mv9@unife.it (S.M.); 2 Department of Life Sciences and Biotechnology, Section of Biochemistry and Molecular Biology, University of Ferrara, Via Fossato di Mortara 74, 44121 Ferrara, Italy; chiara.tupini@unife.it (C.T.); ilaria.lampronti@unife.it (I.L.); 3 Department of Chemical, Pharmaceutical and Agricultural Sciences, University of Ferrara, Via Luigi Borsari 46, 44121 Ferrara, Italy; alessandra.rizzo@unife.it

**Keywords:** benzothiazole derivatives, multifunctional drugs, antioxidant, sunscreen UV-filter, melanoma

## Abstract

Multifunctional drugs represent an emerging strategy for treating complex skin disorders and melanoma. A series of benzothiazole-based hybrids incorporating gallic and syringic acid moieties was synthesized and evaluated as multifunctional agents for skin-related applications. Six hydrazone (**GAHYDR1–3**) and acyl-hydrazone (**GACIN1–3**) derivatives were obtained and fully characterized. Hydroxylated compounds showed the strongest antioxidant activity, with **GAHYDR1** and **GACIN1** displaying low DPPH IC_50_ values and high FRAP reducing power. UV–Vis studies revealed strong UVA–UVB absorption, with molar extinction coefficients comparable to or exceeding those of PBSA. Photoprotective evaluation showed SPF values up to 10.09 (**GACIN2**) and broad-spectrum behavior for selected derivatives. Antioxidant activity remained substantially stable over 3 months in solution. Antiproliferative assays against Colo38, A375, and HaCaT cell lines indicated generally low cytotoxicity toward non-tumor cells. Notably, **GAHYDR3** exhibited selective activity against A375 melanoma cells (IC_50_ = 8.75 µM; SI = 8.12). Overall, phenolic substitution emerged as a key determinant of biological activity, highlighting hydroxylated benzothiazole hybrids as promising antioxidant and photoprotective agents, with **GAHYDR3** representing a potential lead for anti-melanoma development.

## 1. Introduction

The term “multifactorial diseases” refers to conditions with a complex, heterogeneous etiology resulting from interactions among genetic, environmental, and lifestyle factors. These diseases manifest with a wide range of symptoms and involve numerous and diverse biological targets. Significant examples of multifactorial diseases include cancer, Alzheimer’s disease, Parkinson’s disease, diabetes, cardiovascular diseases, central nervous system (CNS) disorders, MAFLD/metabolic disease, and rheumatoid arthritis [1,2,3,4,5].

In response to the complexity of these conditions, there has been growing interest in multifunctional drugs. These are single entities with multiple pharmacological actions, capable of simultaneously interacting with different molecular targets or receptors. The multifunctional approach represents an innovative and effective therapeutic strategy, offering significant advantages such as a reduced need for combination therapy, lower incidence of drug–drug interactions, and a more favorable pharmacokinetic and pharmacodynamic profile [6,7,8,9].

Benzothiazole and its derivatives represent a class of heterocyclic compounds characterized by a thiazole ring fused to a benzene ring. This core structure, defined as 1,3-benzo[d]thiazole, is widely recognized as a chemical scaffold for drug design. A key feature relevant to biological activity, particularly anticancer activity, is the molecule’s planar structure, which facilitates interaction with DNA. The aryl substitution at the 2-position of the benzothiazole ring is considered crucial for both the anticancer properties and the solubility of the compounds [10,11,12].

The versatility of benzothiazole as a scaffold lies in its ability to introduce different substituent groups at various positions on the ring, yielding derivatives with a broad spectrum of biological activities, including antitumor, antimicrobial (antibacterial and antifungal), anti-inflammatory, analgesic, anti-HIV, antioxidant, anticonvulsant, antitubercular, antidiabetic, antihistamine, antimalarial, and neuroprotective properties, with particular interest in neurodegenerative diseases such as Alzheimer’s disease [8,9,10,13,14,15,16,17]. Benzothiazole is not merely a carrier of biological activity; its adaptability makes it an ideal candidate for the development of multifunctional drugs. Research continues to explore how targeted structural modifications, such as the introduction of phenolic groups or hybridization with other molecules, can further optimize the pharmacological properties of benzothiazole derivatives, making this class of compounds a strategic element in the design of future multifactorial therapies [18,19,20,21].

Gallic acid, or 3,4,5-trihydroxybenzoic acid, is a naturally occurring phenolic compound widely found in numerous plants, fruits, and common foods. Its molecular structure is characterized by the presence of three hydroxyl groups (-OH) on the aromatic ring and a carboxyl group (-COOH), which confer significant versatility to the compound. This conformation allows it to interact with various biological targets and exhibit a wide range of pharmacological activities. For these reasons, gallic acid is considered a promising lead compound for the development of new therapeutic agents [22,23,24].

One of the main and most studied activities of gallic acid is its antioxidant action. Its efficacy in eliminating free radicals (ROS scavenging) and in counteracting oxidative damage to lipids, DNA, and proteins is intrinsically linked to its three hydroxyl groups and the carboxyl group [25,26].

Gallic acid and its derivatives have shown promising antioxidant activity and anticancer potential against a wide variety of human cancer cell lines, including those of lung, prostate, breast, colon, cervical, leukemia, hepatocellular carcinoma (HepG2), melanoma (Colo-38), and glioblastoma [22,27,28,29,30].

The structure of syringic acid corresponds to that of a phenolic acid consisting of a benzene ring to which a carboxyl group (-COOH), a hydroxyl group (-OH) at the 4-position, and two methoxyl groups (-OCH_3_) at the 3- and 5-positions are attached. Many of the therapeutic properties attributed to syringic acid are primarily due to its methoxy groups, while the hydroxyl group contributes significantly to its antioxidant activity, particularly its ability to scavenge free radicals [31,32].

In addition to its antioxidant activity, syringic acid also exhibits significant anti-inflammatory effects, due to its ability to modulate the signaling pathways involved in inflammatory processes, including, in particular, the NF-κB pathway, which regulates the expression of numerous pro-inflammatory genes [33,34,35].

This acid has also demonstrated a potential antitumor effect, as it is capable of inhibiting the growth and proliferation of various tumor cell lines, implying a possible use as a chemopreventive agent in cancer therapy [32]. In particular, syringic acid has shown excellent inhibitory effects on UV-induced skin carcinogenesis, suggesting that it may be highly beneficial in preventing skin carcinogenesis [36].

**Figure 1:**
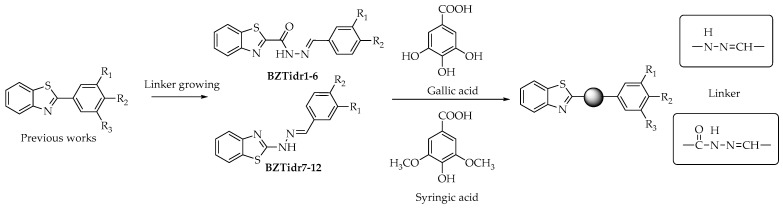
Rational chemical drug design.

Based on these findings, and as a continuation of our works, here we report and discuss the results of a study aimed to complete the structure-activity relationship of two previously investigated series, benzothiazole hydrazone and acyl-hydrazone derivatives (Figure 1) [9,37], by introducing via gallic and syringic acid derivatives, three methoxy and/or hydroxy substituents on the phenolic moiety of the molecule. The aim is to obtain multifunctional compounds for use in the treatment of skin-related diseases; each compound was investigated to determine its antioxidant, UV-filtering, and antiproliferative activity.

## 2. Results and Discussion

### 2.1. Chemistry

In this study, six benzothiazole hybrid molecules were synthesized by incorporating hydrazone (**GAHYDR1–3**) and acyl-hydrazone (**GACIN1–3**) linkers into gallic and syringic acid derivatives.

The three hydrazone derivatives were obtained by following and adapting a previously reported procedure [38].

**Scheme 1:**
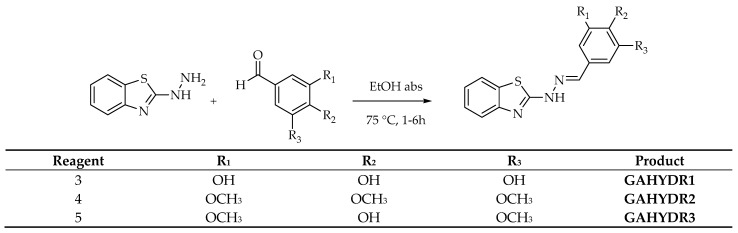
Synthesis and reagents of compounds **GAHYDR1–3**.

In summary, for the **GAHYDR1–3** series, 2-hydrazinobenzo[d]thiazole (**1**) was reacted in EtOH abs with the corresponding aldehyde (Scheme 1). After the starting materials had completely dissolved at room temperature, the reaction was refluxed at 75 °C for a period ranging from 1 to 6 h with the aim of obtaining the compounds with the highest degree of purity.

**Scheme 2:**
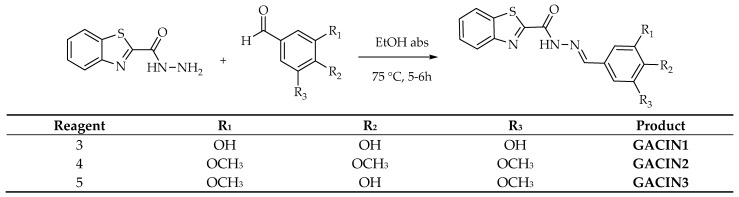
Synthesis and reagents of compounds **GACIN1–3**.

The acylhydrazone derivatives **GACIN1–3** were obtained starting from benzo[d]thiazole-2-carboxydrazide (**2**), which was reacted with the corresponding aldehyde (Scheme 2) in EtOH abs. After the starting reagents had completely dissolved at room temperature, the reaction was refluxed at 75 °C for 3 to 6 h to obtain the **GACIN1–3** compounds with the highest degree of purity.

### 2.2. Antioxidant Profile

#### 2.2.1. DPPH, FRAP, and PCL Tests

The assessment of antioxidant activity is a constantly evolving field, due to the wide variety of compounds with this potential and the multiple mechanisms through which they can act. Over the years, many methods have been developed that differ in numerous characteristics—including the radical-scavenging reaction mechanism or the chemical reagents used—to enable a broad-spectrum evaluation [39,40,41].

For this reason, the antioxidant activity of the synthesized compounds was assessed using three different antioxidant assays: the 2,2-diphenyl-1-picrylhydrazyl (DPPH) radical scavenging activity, the ferric reduction antioxidant power (FRAP), and Photochemoluminescence (PCL) (Table 1).

The DPPH assay measures the antioxidant capacity of a sample against 2,2-diphenyl-1-picrylhydrazyl (DPPH•), a highly stable, commercially available nitrogen-containing radical characterized by a deep purple color. When an antioxidant (capable of transferring a hydrogen atom to the radical) is added to a DPPH solution, the radical (DPPH•) is reduced to its non-radical form, which is pale yellow or colorless (DPPH-H). This bleaching is directly proportional to the sample’s ability to scavenge free radicals.

All synthesized compounds were initially tested against the DPPH radical at a concentration of 1 mg/mL to assess their preliminary profile in terms of the percentage of radical inhibition. Compounds that showed an inhibition percentage greater than 50% at a concentration of 1 mg/mL were further investigated to determine their IC_50_ value expressed in μg/mL.

The results of the initial DPPH screening showed that most of the compounds exhibited good antioxidant activity; however, the most promising compounds were **GAHYDR1**, **GAHYDR2**, **GACIN1**, and **GACIN3**, which exhibited inhibition percentages exceeding 70% at the tested concentration. The IC_50_ values highlighted the importance of the substituents. It was observed that compounds with hydroxyl groups as substituents have lower IC_50_ values and thus greater antioxidant efficacy (**GAHYDR1** and **GACIN1** with IC_50_ values of 8.09 and 9.27 µg/mL, respectively, *p* = 0.2817). The **GAHYDR2** derivative, which has three methoxy substituents, showed the highest value (26.41 µg/mL) among the compounds examined and thus the lowest activity. The **GACIN3** derivative, on the other hand, showed a good IC_50_ value (9.83 µg/mL), likely influenced by both the type of linker present in the molecule and the presence of a hydroxyl group among the substituents.

These data clearly demonstrated the importance of the linkers, as compounds with the same substituents exhibited different activity profiles: this was confirmed by comparing the two derivatives with all methoxy substituents (**GAHYDR2** and **GACIN2**) and those with syringic acid substituents (**GAHYDR3** and **GACIN3**).

The substituents present on the aromatic ring were found to be of fundamental importance for antioxidant activity. In particular, it was observed that hydroxyl groups contributed significantly to the activity, as evidenced by the comparison with syringic acid, which has only one, and with methoxy derivatives, which lack them.

The DPPH data obtained from the hydrazone and acyl-hydrazone derivatives, in terms of structure-activity relationships, showed that the presence of three hydroxyl groups on the aromatic ring, regardless of the linker, did not improve the antioxidant profile. In fact, the two 3,4-dihydroxy-substituted derivatives (**BZTidr4** and **BZTidr10**) are those with the lowest IC_50_ value (3.27 and 4.66 µg/mL, respectively, *p* = 0.0030 and *p* = 0.0047 compared to **GACIN1** and **GAHYRD1**), demonstrating that the catechol function is responsible for the high antioxidant activity, attributable to the ability to form a resonance-stabilized radical.

The Ferric Reducing Antioxidant Power (FRAP) assay is a widely used method for assessing the antioxidant capacity of biological and food samples [39,40,42].

The purpose of this test is to evaluate an antioxidant’s ability to act as a reducing agent, specifically its ability to donate electrons and reduce a ferric complex (Fe^3+^) to a ferrous complex (Fe^2+^) in an acidic environment, specifically at pH 3.6.

The FRAP assay further highlighted how the influence of substituents is crucial for achieving a good antioxidant profile. **GAHYDR1** and **GACIN1** derivatives, characterized by the presence of three hydroxyl groups, exhibited higher activity, with similar values of 8850.41 and 8781.68 μmol TE/g, respectively (*p* = 0.9510). **GAHYDR2** and **GACIN2** derivatives, characterized by the presence of three methoxy substituents, are the molecules with the lowest activity, while **GAHYDR3** and **GACIN3** derivatives, which have a single hydroxyl group, exhibit intermediate activity.

Compared to the catechol derivatives, no difference was observed between the two hydrazone compounds **GAHYDR1** and **BZTidr10** (*p* = 0.4057), whereas among the acyl-hydrazone derivatives, **GACIN1** showed a marked improvement in the reducing profile compared to **BZTidr4** (*p* = 0.0178).

The photochemiluminescence (PCL) test is based on the reaction of a specific radical species, the superoxide anion (O_2_•^−^)—which is generated photochemically upon UV irradiation—with a compound capable of emitting chemiluminescence. The antioxidant activity evaluated by PCL highlighted a structure–activity relationship partially different from that observed in the DPPH and FRAP assays. Among the tested compounds, **GAHYDR3** and **GACIN3** exhibited the highest antioxidant capacities, with PCL values of 54,875.00 and 15,250.00 µmol TE/g, respectively, whereas the fully hydroxylated (**GAHYDR1**, **GACIN1**) and fully methoxylated (**GAHYDR2**, **GACIN2**) derivatives displayed lower activities.

This trend suggests that the simultaneous presence of one phenolic hydroxyl group and two methoxy substituents provides the most favorable balance for scavenging superoxide radicals generated under PCL conditions. The hydroxyl group may contribute directly through hydrogen-atom donation, while the methoxy groups can modulate the electron density of the aromatic ring, stabilizing the resulting radical species and enhancing antioxidant effectiveness.

Interestingly, the PCL results do not fully correlate with those obtained by DPPH and FRAP assays, where the trihydroxylated derivatives **GAHYDR1** and **GACIN1** showed the strongest antioxidant profile. This discrepancy can be explained by the different mechanisms involved in the assays. While DPPH and FRAP mainly evaluate hydrogen atom transfer and reducing power, respectively, PCL specifically measures the ability of compounds to quench superoxide radicals under photochemical conditions.

From a structure–activity relationship perspective, these findings indicate that antioxidant activity is strongly dependent on the nature of the reactive species considered. Whereas multiple hydroxyl groups favor radical scavenging and ferric ion reduction, the syringic acid-derived substitution pattern appears particularly effective against superoxide radicals. Moreover, the comparable behavior observed for **GAHYDR3** and **GACIN3** suggests that, under PCL conditions, the substitution pattern exerts a greater influence on activity than the nature of the hydrazone or acylhydrazone linker.

**Figure 2:**
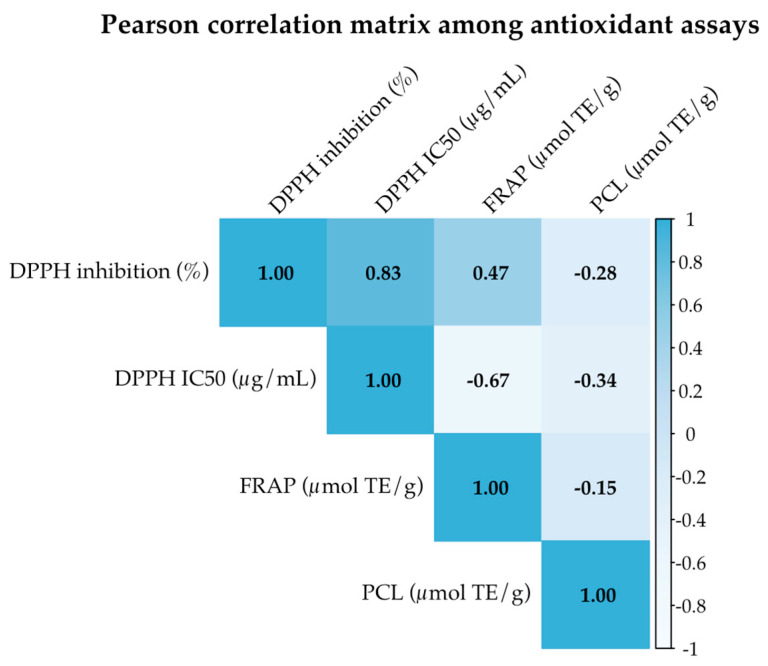
Correlation matrix showing the relationships among DPPH, FRAP, and PCL assays.

To further investigate the relationships among the antioxidant assays, a Pearson correlation analysis was performed using the values obtained from the DPPH inhibition, DPPH IC_50_, FRAP, and PCL measurements (Figure 2). A strong positive correlation was observed between DPPH inhibition and antioxidant performance derived from DPPH IC_50_ (r = 0.83), confirming the internal consistency of the DPPH assay. Furthermore, FRAP values showed a moderate positive correlation with DPPH inhibition (r = 0.47) and a moderate negative correlation with DPPH IC_50_ (r = −0.67), indicating that compounds exhibiting greater radical-scavenging activity generally also possessed greater reducing power.

In contrast, PCL values showed weak negative correlations with DPPH inhibition (r = −0.28), the DPPH IC_50_ (r = −0.34), and FRAP (r = −0.15). These results support the different trends observed experimentally and suggest that the antioxidant response measured by PCL is governed by mechanisms distinct from those assessed by DPPH and FRAP. While the latter assays primarily assess hydrogen atom transfer and electron-donating capacity, respectively, the PCL specifically measures the elimination of photochemically generated superoxide radicals.

Overall, the correlation analysis confirms that the antioxidant activity of this series of benzothiazole derivatives depends strongly on the analytical method used and highlights the importance of employing complementary methods to obtain a comprehensive characterization of their antioxidant profile.

#### 2.2.2. Stability Study in Solution

The six synthesized derivatives were stored in solution at 4 °C in the dark to assess their stability, measured in terms of the maintenance of antioxidant capacity using the FRAP assay. Table 2 shows the data obtained after 1 month (T30) and after 3 months (T90), compared with the values at T0.

Analyzing the antioxidant data in terms of stability, after one month, a statistically significant decrease in activity (*p* = 0.0071) was observed only for the compound **GAHYDR3**.

After 3 months, **GAHYDR3** showed an activity profile consistent with that observed at 30 days, indicating stabilization: this result highlights an initial reduction in antioxidant capacity that may be related to rapid degradation of the compound in solution, which will be further investigated. The compound **GACIN1** exhibits behavior similar to that of **GAHYDR3**, although the antioxidant activity values at T0 and T1 months are not statistically different (*p* = 0.0255), whereas they are different between T0 and T3 months, highlighting an actual decline in antioxidant activity. In this case, a precipitate was observed in the stored stock solution, which apparently returned to solution following ultrasonic treatment; this finding could therefore be attributed to incomplete re-dissolution, as well as to actual degradation, which is why it warrants further investigation. After 3 months, the compounds **GAHYDR2** and **GACIN2** showed statistically significant increases in FRAP antioxidant activity (*p* = 0.0016 and *p* = 0.0008, respectively), accompanied by a slight hyperchromic effect at λmax (Figure 2), which warrants further investigation.

### 2.3. Evaluation of Filtering Properties

#### 2.3.1. UV-Filtering Parameters in Solution

The spectra of the compounds (**GAHYDR1–3** and **GACIN1–3**) were recorded in solution (5.38–6.64 × 10^−5^ M) in the UVA-UVB range (290–400 nm) to evaluate their absorption profiles and compare them with that of PBSA, a commercial UVB filter with a λmax of 304 nm.

**Figure 3:**
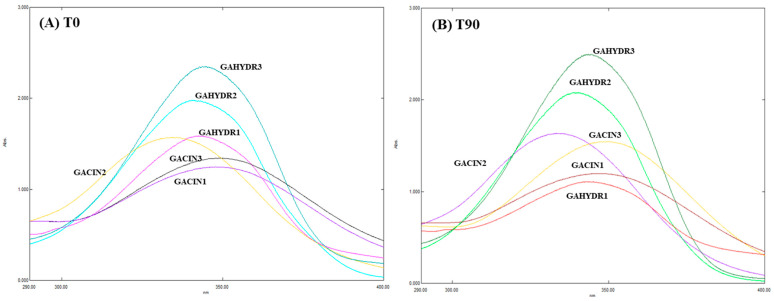
UV spectra of the hydrazone derivatives **GAHYDR1–3** and acylhydrazone derivatives **GACIN1–3** in solution, recorded at time zero ((**A**), T0) and after 3 months (4 °C, dark) ((**B**), T90).

Analysis of the zero-time spectra (Figure 3A) shows that differences in the linkers resulted in different absorption profiles. For the **GAHYDR1–3** series, it was evident that the average absorbance values were higher than those of the **GACIN1–3**, in line with the profile of the two catechol derivatives **BZTidr10** and **BZTidr4**.

When considering only the first series of compounds (**GAHYDR1–3**), it was observed that these derivatives exhibit an almost identical absorption profile, with very similar λmax values (Table 3). In this series, the type and number of substituents appear to influence not so much the absorption profile (and thus the λmax) as the absorption intensity.

When considering ε, it can be observed that all the compounds examined showed values higher than the reference UVB filter (PBSA); therefore, all compounds in the **GAHYDR1–3** series exhibited good UV radiation absorption activity.

An analysis of the values for the acyl-hydrazone series (Table 3) revealed that the compounds containing at least one hydroxyl group (**GACIN1** three -OH groups, and **GACIN3** two -OMe groups and one -OH group) exhibit similar λmax values. The compound **GACIN2**, on the other hand, having three methoxy groups as substituents, exhibited an isochromatic effect.

The data obtained from ε showed that, when compared with PBSA, **GACIN2** and **GACIN3** exhibit good photoprotective properties, while **GACIN1** has a lower molar extinction coefficient than the reference standard.

A comparison of the two series clearly showed that both the linker and the substituents influenced the absorption profile. In particular, regarding the hydrazone series (**GAHYDR1–3**), the absorption profiles of the three derivatives were nearly identical, varying only in terms of absorbance: this suggests a greater (inversely proportional) influence of the different number of hydroxyl substituents on the aromatic ring compared to the linker. The absorption profile of the acyl-hydrazone series compounds, on the other hand, showed the influence of both the number of -OH groups and the linker: the **GACIN2** derivative (3 methoxy substituents), in fact, has an absorption profile different from that of **GACIN1** and **GACIN3**, both in terms of λmax and absorbance.

#### 2.3.2. Photostability Study in Solution

The six derivatives in solution were stored at 4 °C in the dark in order to assess their stability, measured both in terms of the maintenance of antioxidant capacity and in terms of changes in the UV profile.

As can be seen in Figure 3B, the most significant findings concerned the compounds **GAHYDR1** and **GACIN1**, which after 3 months showed a clear hypochromic effect at λmax (which, however, did not change).

For both compounds, a precipitate was observed in the stored stock solution, which apparently returned to solution following ultrasonic treatment.

In the case of **GACIN1**, the hypochromic effect was associated with a statistically significant decrease in antioxidant activity; this finding could therefore be attributed to incomplete solubilization or actual degradation, which is why it warrants further investigation.

In contrast, the hypochromic effect of **GAHYDR1** was not associated with any decrease in antioxidant activity.

The UV profile of **GAHYDR3** in solution, after 3 months, showed no changes that could be attributed to the decrease in antioxidant activity observed in the FRAP assay.

In general, we can state that none of the compounds, after 3 months in solution, showed changes in λmax; therefore, no bathochromic or isochromic effect was observed for any of them. The variations concern the intensity of absorption, specifically a hyperchromic effect for **GAHYDR2**, **GAHYDR3**, **GACIN2**, and **GACIN3**, and a hypochromic effect for **GAHYDR1** and **GACIN1**; these are not always correlated with an increase or decrease in antioxidant activity and therefore require further investigation.

#### 2.3.3. In Vitro Photoprotective Activity in Solutions

The demand for safer and more effective sunscreen ingredients has spurred research efforts aimed at discovering new organic UV filters. The focus has been on molecules capable of providing broad-spectrum protection, with particular attention to the long-wave UVA range [43].

Determining the SPF (Sun Protection Factor) value in solution is one of the in vitro methods used to predict the potential effectiveness of sunscreens. It involves a spectrophotometric measurement of UV transmittance, and the indicative SPF value is calculated using Equation (1):

(1) $$SPF=\;\frac{\sum_{290}^{400}E_{\lambda }S_{\lambda }\Delta_{\lambda }}{\sum_{290}^{400}E_{\lambda }S_{\lambda }T_{\lambda }\Delta_{\lambda }}$$

where Eλ corresponds to the erythemal spectrum of sunlight, Sλ represents the spectral irradiance of sunlight, Tλ corresponds to the spectral transmittance of a product, and ∆λ is the wavelength step equal to 1 nm.

The European Commission’s recommendation of 22 September 2006, on the efficacy of sunscreen products (2006/647/EC) [44] sets out the minimum efficacy requirements for a sunscreen product, which include an SPF value of 6 or higher, a UVA/UVB ratio of 1:3, and a critical wavelength (λc) greater than 370 nm. According to the U.S. Food and Drug Administration (21 CFR Parts 347 and 352, Sunscreen Drug Products for Over-the-Counter Human Use) [45], a λc value greater than 370 nm ensures broad-spectrum sun protection, capable of simultaneously protecting the skin from both UVA and UVB rays.

As a preliminary analysis, the determination of the filtering parameters was performed for all compounds in solution at a concentration of 5.38–6.64 × 10^−5^ M, starting from a 1 mg/mL stock solution in methanol. The UV spectra of the compounds under examination were recorded between 290 and 400 nm—a range encompassing both UVA and UVB—using a SHIMADZU UV-2600 spectrophotometer, and compared with those of the PBSA reference UVB filter. Using the SPF CALCULATOR software, the values shown in Table 4 were obtained from the spectra.

Analysis of the results showed that all compounds exhibited superior characteristics, in every respect, compared to the reference PBSA compound.

As noted above, a broad-spectrum compound must have a critical λ of 370 nm or greater. This parameter holds true for four of the compounds obtained, **GAHYDR1**, **GACIN1**, **GACIN2**, and **GACIN3**, highlighting how the type of linker influences the evaluation and determination of this type of activity.

Among the compounds analyzed, the one with the highest SPF value was **GACIN2** (10.09), characterized by an acyl-hydrazone linker and all methoxy substituents. It can be hypothesized that this compound provides good protection against both UVA and UVB rays, considering also the value of the UVA/UVB ratio (1.75).

The UVA/UVB ratio of all the synthesized compounds, which should be at least 1/3, was found to be greater than 1.75, thus indicating greater absorption in the UVA region.

In terms of structure-activity relationships, it was observed that, for both hydrazone and acyl-hydrazone derivatives, the presence of three substituents on the aromatic ring led to an improvement in all filtering parameters in solution compared to the two catechol derivatives, **BZTidr10** and **BZTidr4**.

These promising results will be further investigated by incorporating the derivatives with the best safety profile into dermo-cosmetic formulations for sun protection to verify the maintenance of the filtering parameters.

### 2.4. Antiproliferative Activity on Colo38, A375, and HaCaT Cell Lines

In order to evaluate their potential antiproliferative efficacy, all synthesized compounds were tested in vitro across three different cell lines. The immortalized human HaCaT keratinocytes were used as a control to assess cytotoxicity against non-tumor skin cells and thus determine selectivity toward tumor cell lines. The human melanoma Colo38 and the A375 cell lines were chosen as skin tumor models. While the A375 cell line serves as the gold standard for investigating the cellular biology and pharmacology of primary melanoma, Colo38 represents a valuable metastatic model for cancer immunology. The data are summarized in Table 5: antiproliferative activity is expressed as the IC_50_ value in μM; the selectivity index (SI) was also calculated, indicating the ratio between the IC_50_ of a given compound against the non-cancerous cell line and the IC_50_ of the same compound against the tumor cell line. Cisplatin was used as a positive control for the HaCat cell line, while a previously evaluated benzimidazolohydrazone derivative (Cmp 13) [46] was used as a positive control for the Colo38 cell line.

Compounds belonging to the hydrazone and acylhydrazone series, with the exception of **GAHYDR1**, exhibited inhibitory effects on the growth of non-tumor cell lines at high concentrations, between 40 and 90 μM. Regarding activity against Colo38, the derivatives **GAHYDR1**, **GACIN1**, and **GACIN2** were the only ones to exhibit IC_50_ values below 100 μM: **GACIN2** with a selectivity index below 1, while **GAHYDR1** and **GACIN1** had SI above 1, though still not considered active potential anticancer candidates.

The derivatives of **GAHYDR1** and **GACIN1**, both bearing three hydroxyl groups, exhibited the best antioxidant profile (as reported above), demonstrating their activity against the DPPH radical with IC_50_ values of 8.09 and 9.27 µg/mL, respectively: these concentration values (corresponding to 27 and 28 μM, respectively, when expressed in molar concentration) are well below the toxicity threshold observed in healthy keratinocyte lines (>100 μM and 80.64 μM, respectively), indicating their potential for use in topical formulations.

The results of the antiproliferative assays about the Colo38 cell line showed that the introduction of three substituents on the aromatic ring did not result in increased selectivity or a corresponding increase in activity against melanoma cells for either the hydrazone series or the acylhydrazone series. In fact, the best profile remains that of the two catechol derivatives, **BZTidr10** and **BZTidr4**.

It is interesting to note that a different trend emerged in the A375 melanoma cell line. Among all the derivatives tested, **GAHYDR3** showed the most promising antiproliferative profile, with an IC_50_ value of 8.75 ± 1.48 μM and a selectivity index of 8.12, indicating a marked preference for melanoma cells over non-tumorous HaCaT keratinocytes. In contrast, all other compounds tested against A375 exhibited weak activity (IC_50_ > 80 μM) or no significant growth inhibition (IC_50_ > 100 μM). These results suggest that, although the overall structure-activity relationship did not reveal a general improvement associated with the replacement of the aromatic ring, the **GAHYDR3** scaffold could represent an interesting starting point for further optimization aimed at targeting primary melanoma cells. In addition, a preliminary SwissADME analysis of **GAHYDR3** revealed favorable drug-likeness and pharmacokinetic properties, including compliance with Lipinski, Veber, Egan, and Ghose criteria, supporting its potential as a promising lead for further anti-melanoma optimization (Table S1) [47].

## 3. Materials and Methods

### 3.1. General

All reagents were purchased from Sigma-Aldrich S.r.l., Milano, Italy, and Acros Organics, Geel, Belgium. All solvents used were purchased from Carlo Erba Reagents SRL, Milano, Italy, and used without further purification. Silica gel plates were used to perform TLC analysis (Macherey-Nagel Poligram SIL G/UV2540.20 mm, GmbH and Co. KG Neumann-Neander-str. 6–8, Dueren, Germany) and visualized by a UV lamp with the wavelength fixed to 254 nm and/or with a solution of KMnO_4_ (1%). Molecular weights were determined by ESIMS (Micromass ZMD 2000, Waters, Milford, MA, USA), and the values are reported as [M + H]^+^. Melting points were determined by a Stuart melting point apparatus. ^1^H-NMR and ^13^C-NMR were registered on the VXR-200 Varian spectrometer (Palo Alto, CA, USA) at 200 MHz and 400 MHz, using TMS (Tetramethylsilane) as an internal standard. The chemical shift in each signal is expressed as units δ (ppm) relative to the signal of deuterated solvents used (DMSO-*d*_6_ and CDCl3). The following abbreviations are used to designate multiplicity and assignment: s (singlet), d (doublet), t (triplet), m (multiplet), dd (double doublet), BZT (benzothiazole), and Ar (Aryl). UV spectrophotometric analysis was conducted on a UV-Vis spectrophotometer (UV-31 SCAN ONDA Spectrophotometer, Giorgio Bormac spectrophotometer Srl, Carpi (MO), Italy) or on a UV-Vis spectrophotometer (Shimadzu UV-2600, Nakagyo-ku, Japan).

### 3.2. Chemistry

#### 3.2.1. Synthesis of 2-Hydrazineylbenzo[d]thiazole (**1**)

To a solution of benzo[d]thiazole-2-thiol (1 eq) in absolute EtOH abs (7 mL) in a round-bottomed flask, 25% *w*/*w* hydrazine hydrate (4 eq) was added, and the reaction was refluxed at 75 °C for 48 h. The reaction was monitored by TLC, and the evolution of H_2_S was detected with a lead acetate paper. The solution was allowed to cool to room temperature, during which crystals formed, and was filtered through a Gooch filter, washing with cold deionized water. Finally, it was left to dry in an oven overnight. A white powder was obtained as the desired product with an excellent yield (91.7%). Analytical data are in agreement with those reported in the literature [48].

#### 3.2.2. Synthesis of Benzo[d]thiazole-2-carbohydrazide (**2**)

To a solution of benzo[d]thiazole-2-etylcarboxylate (1 eq) in EtOH abs (5 mL) in a 50 mL round-bottomed flask, a 25% *w*/*w* solution of hydrazine hydrate (3 eq) was added. The mixture was heated to reflux conditions for 1 h. After completion, when the spot of the starting material was no longer visible on TLC, the solution was allowed to warm to room temperature. The needle-shaped crystal solid formed during cooling was separated on a Gooch filter and washed several times with cold deionized water, and then dried in an oven overnight. The pale powder yield was 78.8%. Analytical data agree with those reported in the literature [9,49].

#### 3.2.3. General Procedure for the Synthesis of Compounds **GAHYDR1–3**

In a flask with a neck of suitable size, compound **1** (2-hydrazinobenzo[d]thiazole) (1 eq) and EtOH abs (5 mL) were added, and the mixture was stirred at room temperature until completely dissolved (15 min). The appropriate aldehyde (**3**, **4**, **5**) (1 eq) was then added, and the reaction was refluxed at 75 °C for 1–6 h.

The reaction was monitored by TLC: upon completion, the solvent was removed, and water was added to the resulting solid, which was then extracted with ethyl acetate. The mixture was dried over Na_2_SO_4_, and the organic phase was concentrated and crystallized by the hot-cold method (acetone/water). The product was filtered on Gooch and dried in an oven overnight to afford target compounds **GAHYDR1–3** in good yields.

##### (E)-5-((2-(Benzo[d]thiazol-2-yl)hydrazinylidene)methyl)benzene-1,2,3-triol (**GAHYDR1**)

Light-sensitive brown solid, yield: 58.0%. m.p. 165 °C. ^1^H NMR (400 MHz, DMSO-*d*_6_) δ 11.91 (s, 1H, BTZ–NH–N), 9.08 (s, 2H, OH), 8.54 (s, 1H, OH), 7.84 (s, 1H, Ar–CH=), 7.73 (dd, *J* = 7.9, 1.2 Hz, 1H, BTZ H), 7.39 (d, *J* = 8.1 Hz, 1H, BTZ H), 7.32–7.21 (m, 1H, BTZ H), 7.14–6.95 (m, 1H, BTZ H), 6.64 (s, 2H, Ar H).

^13^C NMR (101 MHz, DMSO-*d*_6_) δ 167.12, 146.63, 145.77, 145.69, 136.00, 126.41, 124.98, 121.87, 117.82, 109.19, 106.40. ESI-MS[M + H]^+^: calculated for C_14_H_11_N_3_O_3_S, 302.05; found, 302.23.

##### (E)-2-(2-(3,4,5-Trimethoxybenzylidene)hydrazinyl)benzo[d]thiazole (**GAHYDR2**)

White solid, yield: 84.6%. m.p. 204 °C. ^1^H NMR (400 MHz, DMSO-*d*_6_) δ 12.23 (s, 1H, BTZ–NH–N), 8.04 (s, 1H, Ar–CH=), 7.75 (d, *J* = 7.9 Hz, 1H, BTZ H), 7.40 (d, *J* = 8.0 Hz, 1H, BTZ H), 7.30–7.23 (m, 1H, BTZ H), 7.07 (td, *J* = 7.5, 1.2 Hz, 1H, BTZ H), 7.00 (s, 2H), 3.82 (s, 6H, -OCH_3_), 3.68 (s, 3H -OCH_3_). ^13^C NMR (101 MHz, DMSO-*d*_6_) δ 167.48, 153.63, 139.27, 130.35, 128.02, 126.38, 122.52, 122.00, 104.20, 60.57, 56.30. ESI-MS[M + H]^+^: calculated for C_17_H_17_N_3_O_3_S, 344.10; found, 344.18.

##### (E)-4-((2-(Benzo[d]thiazol-2-yl)hydrazinylidene)methyl)-2,6-dimethoxyphenol (**GAHYDR3**)

Opaque white powder that is sensitive to light, yield 83.5%. m.p. 244 °C. ^1^H NMR (400 MHz, DMSO-*d*_6_) δ 12.06 (s, 1H, BTZ–NH–N), 8.83 (s, 1H, Ar–OH), 8.00 (s, 1H, BTZ H), 7.74 (d, *J* = 7.8 Hz, 1H, Ar–CH=), 7.39 (d, *J* = 8.1 Hz, 1H, BTZ H), 7.26 (ddd, *J* = 8.1, 7.3, 1.3 Hz, 1H, BTZ H), 7.06 (td, *J* = 7.5, 1.2 Hz, 1H, BTZ H), 6.96 (s, 2H, Ar H), 3.80 (s, 6H, OCH_3_). ^13^C NMR (101 MHz, DMSO-*d*_6_) δ 148.69, 148.61, 138.11, 137.97, 126.34, 125.86, 125.10, 122.22, 121.97, 121.80, 104.57, 56.40. ESI-MS[M + H] ^+^: calculated for C_16_H_15_N_3_O_3_S, 330.08; found, 330.12.

#### 3.2.4. General Procedure for the Synthesis of Compounds **GACIN1–3**

In a flask with a neck of suitable size, compound **2** (benzo[d]thiazole-2-carbohydrazide) (1 eq) and EtOH abs (5 mL) were added, and the mixture was stirred at room temperature until completely dissolved (15 min). The appropriate aldehyde (**3**, **4**, **5**) (1 eq) was then added, and the reaction was refluxed at 75 °C for 5–6 h.

The reaction was monitored by TLC: upon completion, the solvent was removed, and water was added to the resulting solid, which was then extracted with ethyl acetate. The mixture was dried over Na_2_SO_4_, and the organic phase was concentrated and crystallized by the hot-cold method (acetone/water). The product was filtered on Gooch and dried in an oven overnight to afford target compounds **GACIN1–3** in good yields.

##### (E)-N′-(3,4,5-Trihydroxybenzylidene)benzo[d]thiazole-2-carbidrazide (**GACIN1**)

Light-sensitive yellow powder, yield 60.0%. m.p. 323 °C. ^1^H NMR (400 MHz, DMSO-*d*_6_) δ 12.37 (s, 1H, -COO-NH-), 9.04 (s, 3H, OH Ar), 8.38 (s, 1H, -N=CH- Ar), 8.20 (dd, *J* = 32.1, 8.0 Hz, 2H, BTZ), 7.62 (dt, *J* = 21.4, 7.5 Hz, 2H, BTZ), 6.70 (s, 2H, Ar). ^13^C NMR (101 MHz, DMSO-*d*_6_) δ 164.55, 156.16, 153.22, 151.88, 151.75, 146.68, 136.73, 136.48, 127.71, 127.49, 124.71, 124.48, 123.52, 107.06. ESI-MS[M + H]^+^: calculated for C_15_H_11_N_3_O_4_S, 330.05; found, 330.24.

##### (E)-N′-(3,4,5-Trimethoxybenzylidene)benzo[d]thiazole-2-carbidrazide (**GACIN2**)

White solid with traces of indigo, yield 83.3%. m.p. 255 °C. ^1^H NMR (400 MHz, DMSO-*d*_6_) δ 12.66 (s, 1H, -COO-NH-), 8.57 (s, 1H, -N=CH- Ar), 8.318–8.17 (m, 2H, BTZ), 7.72–7.54 (m, 2H, BTZ), 7.01 (s, 2H, Ar H), 3.83 (s, 6H, Ar–OCH_3_), 3.70 (s, 3H, Ar–OCH_3_). ^13^C NMR (101 MHz, DMSO-*d*_6_) δ 164.25, 156.58, 153.67, 153.18, 150.88, 140.08, 136.51, 129.94, 127.75, 127.59, 124.55, 123.53, 105.01, 60.60, 56.44. ESI-MS[M + H]^+^: calculated for C_18_H_17_N_3_O_4_S, 372.09; found, 372.13.

##### (E)-N′-(4-Hydroxy-3,5-dimethoxybenzylidene)benzo[d]thiazole-2-carbidrazide (**GACIN3**)

White/green powder, yield 70.0%. m.p. 272 °C. ^1^H NMR (400 MHz, DMSO-*d*_6_) δ 12.49 (s, 1H, -COO-NH-), 9.01 (s, 1H, OH), 8.52 (s, 1H, -N=CH- Ar), 8.28–8.21 (m, 2H, BTZ), 7.69–7.55 (m, 2H, BTZ), 6.97 (s, 2H, Ar H), 3.82 (s, 6H, Ar–OCH_3_). ^13^C NMR (101 MHz, DMSO-*d*_6_) δ 164.47, 153.19, 151.54, 149.01, 148.61, 138.91, 136.49, 127.72, 127.52, 124.66, 124.51, 123.52, 105.36, 56.51. ESI-MS[M + H]^+^: calculated for C_17_H_15_N_3_O_4_S, 358.08; found, 358.24.

### 3.3. Antioxidant Activity Assays

#### 3.3.1. Free-Radical-Scavenging Activity on DPPH

The antioxidant activity of the tested compounds was evaluated using the DPPH radical scavenging method, with slight modifications from previously reported procedures [50]. Briefly, 0.75 mL of each sample solution prepared in methanol (or DMSO/methanol, depending on solubility) was mixed with 1.5 mL of a 0.004% DPPH solution in methanol.

The reaction mixtures were kept in the dark at room temperature for 30 min to ensure completion of the reaction and to prevent light-induced degradation. After incubation, absorbance was measured at 517 nm using a UV-Vis spectrophotometer.

Radical scavenging activity was expressed as percentage inhibition, calculated by comparing the absorbance of the sample with that of a control solution lacking the test compound. All compounds were initially screened at a concentration of 1 mg/mL. Samples showing inhibition values higher than 70% were further analyzed to determine their IC_50_ value (μg/mL), obtained by linear regression analysis. Trolox^®^ was used as the reference standard, while gallic and syringic acids served as positive controls.

#### 3.3.2. Ferric-Reducing Antioxidant Power (FRAP) Assay

The reducing ability of the compounds was determined using the ferric-reducing antioxidant power (FRAP) method, based on the reduction of Fe^3+^ to Fe^2+^ in the presence of the TPTZ ligand. The working FRAP reagent was freshly prepared by combining acetate buffer (0.1 M, pH 3.6), TPTZ solution (10 mM in 40 mM HCl), and ferric chloride (20 mM) in a volumetric ratio of 10:1:1. An aliquot of the sample (0.1 mL) was added to 1.9 mL of the FRAP reagent, and the mixture was incubated at 37 °C for 10 min. After incubation, absorbance was measured at 593 nm, corresponding to the formation of the Fe^2+^–TPTZ complex. Quantitative results were expressed as μmol Trolox equivalents per gram of compound (μmol TE/g), using Trolox^®^ as the calibration standard. Gallic and syringic acids were analyzed under the same conditions as reference compounds.

#### 3.3.3. Photochemoluminescence (PCL) Assay

Photochemiluminescence (PCL) measurements were performed using a Photochem^®^ instrument (Analytik Jena, Leipzig, Germany) following the procedure described by Popov and Lewin [51]. The ACL assay kit was employed to assess the antioxidant capacity of lipophilic constituents. In brief, superoxide radicals were photochemically generated from luminol under UV light exposure (351 nm, 3 mW cm^−2^). Luminescence was monitored over a 180 s period, and the integrated signal was determined as the area under the emission curve using the manufacturer’s software. Results were calculated from a Trolox standard curve and reported as μmol TE g^−1^ dry matter. Before being analyzed in triplicate, the samples were suitably diluted in methanol.

### 3.4. Evaluation of Filtering Parameters in Solution

The samples were dissolved in MeOH at a concentration of 1 mg/mL. All solutions were vortexed and, in some cases, also sonicated to facilitate the dissolution process. For analysis, each compound was diluted in methanol to a final concentration range of 5.38–6.64 × 10^−5^ M.

To analyze the compounds in solution, the absorbance (between 290 and 400 nm) was measured using a spectrophotometer. The data were then processed using SPF CALCULATOR software (SPF Calculator Software version 2.1, Shimadzu, Milan, Italy) to obtain the filtering parameters (SPF, UVAPf, UVA/UVB, and λ_c_).

### 3.5. Cell Growth Inhibition Assays

To evaluate the potential cytotoxicity of compounds, three different cell lines were employed: HaCaT keratinocytes, and the human melanoma cell lines Colo38 and A375. Skin HaCaT keratinocytes cells were obtained by “Istituto Zooprofilattico Sperimentale della Lombardia e dell’Emilia Romagna”, Brescia, Italy. Human melanoma Colo38 cells were kindly provided by Dr. Patrizio Giacomini (Laboratory of Immunology, Regina Elena Institute, Rome, Italy) [9], while A375 cells were recently purchased by the Lampronti research group (AddexBio, San Diego, CA, USA). Cells were seeded and, after 24 h, treated with the compounds at increasing concentrations (0.01 μM, 0.1 μM, 1 μM, and 100 μM). To determine the concentration of compound leading to 50% inhibition of cell growth (IC_50_), experiments were performed in triplicate using narrower concentration around the specific IC_50_ value identified for each formulation. HaCaT cells were maintained in DMEM-F12 medium supplemented with 10% fetal bovine serum (FBS), 1% penicillin/streptomycin, and L-glutamine. Colo38 cells were cultured in RPMI-1640 medium supplemented with 10% FBS, 1% penicillin/streptomycin, and L-glutamine, whereas A375 cells were maintained in DMEM supplemented with 10% FBS, 1% penicillin/streptomycin, and L-glutamine, glucose, NaHCO_3,_ and sodium pyruvate. All cell lines were cultured at 37 °C in a humidified atmosphere containing 5% CO_2_. After 48 h of treatment, cell viability was quantified by Coulter Counter-based cell counting. For cell harvesting, while Colo38 cells were gently detached with a cell scraper, other cultures were gently rinsed with Dulbecco’s phosphate-buffered saline (DPBS) 1X and incubated with 200 µL of trypsin per well for 5–10 min at 37 °C. Trypsin was neutralized by adding 200 µL FBS, followed by 600 µL of complete medium. The resulting cell suspension was collected, and 50 µL of each cell line was diluted in 5 mL of physiological solution prior to counting using a Z2 Coulter Counter (Beckman Coulter, Hialeah, FL, USA) [52,53].

## 4. Conclusions

In this work, a new series of benzothiazole-based hybrid derivatives containing gallic and syringic acid moieties was successfully designed, synthesized, and biologically evaluated, confirming the effectiveness of the molecular hybridization strategy in generating multifunctional compounds. The results clearly demonstrated that both the nature of the linker (hydrazone vs. acyl-hydrazone) and the substitution pattern on the aromatic ring play a crucial role in modulating the biological profile. In particular, the hydroxylated derivatives emerged as the most promising compounds, exhibiting strong antioxidant activity, significant UV-filtering properties, and a favorable safety profile toward non-tumor cells. Although most compounds showed only moderate antiproliferative effects, GAHYDR3 displayed selective activity against A375 melanoma cells, with an IC_50_ value of 8.75 μM and a selectivity index of 8.12, identifying this derivative as a promising lead for further anticancer optimization.

From a structure-activity relationship perspective, phenolic hydroxyl groups—particularly the catechol group—played a crucial role in enhancing antiradical properties, likely due to increased hydrogen atom donation and radical stabilization. Furthermore, the linker modulated both antioxidant efficacy and photoprotective behavior. These results are consistent with the recognized versatility of the benzothiazole core, widely regarded as a privileged scaffold capable of interacting with multiple biological targets and exerting a broad range of pharmacological activities [12,20,54].

It should be noted that the hybrid design approach adopted in this study aligns with current strategies for addressing multifactorial diseases, where the simultaneous modulation of different pathological pathways—such as oxidative stress, UV-induced damage, inflammation, and abnormal cell proliferation—is required. Benzothiazole-based hybrids, due to their structural adaptability and ability to act on multiple targets, represent promising candidates for the development of multifunctional therapeutic agents [19].

Overall, the present study highlights the potential of these hybrid molecules as multifunctional agents for skin-related disorders, combining antioxidant and photoprotective properties with low toxicity toward healthy skin cells. Moreover, the selective antiproliferative activity observed for **GAHYDR3** against A375 melanoma cells suggests that this scaffold warrants further investigation as a potential anti-melanoma lead compound within a multi-target drug design strategy.

## Figures and Tables

**Table 1:** In vitro antioxidant profile of the synthesized compounds. Each value is the mean of at least three different experiments (mean ± SEM).

Compound	DPPH (% Inhibition)	DPPH IC_50_ (µg/mL)	FRAP (µmolTE/g)	PCL (µmolTE/g)
**Gallic acid**	-	1.63 ± 0.04	19,154.56 ± 10.20	8160.00 ± 452.55
**Syringic acid**	-	5.61 ± 0.32	10,590.98 ± 12.30	4600.00 ± 226.27
**GAHYDR1**	73.58 ± 0.10	8.09 ± 0.27	8850.41 ± 638.34	1906.25 ± 8.84
**GAHYDR2**	94.32 ± 0.09	26.41 ± 1.71	1545.90 ± 31.29	2031.25 ± 97.23
**GAHYDR3**	40.18 ± 0.39	-	3768.27 ± 129.37	54,875.00 ± 176.78
**BZTidr10**	-	4.66 ± 0.54	9446.17 ± 65.04	-
**GACIN1**	85.42 ± 0.14	9.27 ± 0.91	8781.68 ± 835.20	8550.00 ± 141.42
**GACIN2**	14.24 ± 0.08	-	201.30 ± 9.83	165.00 ± 4.24
**GACIN3**	72.42 ± 0.00	9.83 ± 0.64	2972.34 ± 160.30	15,250.00 ± 919.24
**BZTidr4**	-	3.27 ± 0.21	5506.15 ± 120.52	-

**Table 2:** FRAP values of hydrazone and acylhydrazone derivatives. The results are the average of at least three separate experiments (mean ± SD). (* *p* < 0.05).

Compound	FRAP T0 (µmolTE/mL)	FRAP T30 (µmolTE/mL)	FRAP T90 (µmolTE/mL)
**GAHYDR1**	8850.41 ± 638.34	7640.94 ± 85.949	8054.66 ± 127.98
**GAHYDR2**	1545.90 ± 31.29	1546.65 ± 63.75	1635.32 ± 10.35 *
**GAHYDR3**	3768.27 ± 129.37	3429.92 ± 17.05 *	3470.86 ± 89.95 *
**GACIN1**	8781.68 ± 835.20	7415.51 ± 94.78	7494.94 ± 112.36 *
**GACIN2**	201.30 ± 9.83	220.5130 ± 0.28	253.43 ± 5.17
**GACIN3**	2972.34 ± 160.30	2969.25 ± 64.56	2968.60 ± 75.69

**Table 3:** Values of λmax and ε for hydrazone and acylhydrazone derivatives.

Compound	λmax (nm)	ε (dm^3^ mol^−1^ cm^−1^)
**GAHYDR1**	342.90	23,900.60
**GAHYDR2**	341.15	34,115.12
**GAHYDR3**	344.25	38,552.63
**BZTidr10**	341.4	39,017.09
**GACIN1**	348.15	23,101.85
**GACIN2**	334.80	28,866.17
**GACIN3**	349.05	24,089.29
**BZTidr4**	346.30	22,183.99
**PBSA**	304.50	23,881.86

**Table 4:** SPF, UVAPF0, critical λ, and UVA/UVB ratio values for hydrazine and acyl-hydrazone derivatives, and PBSA in solution.

Compound	SPF	UVAPF0	λc (nm)	UVA/UVB
**GAHYDR1**	5.50	8.55	373	2.75
**GAHYDR2**	6.81	9.35	367	3.62
**GAHYDR3**	7.01	13.57	369	4.76
**BZTidr10**	3.73	4.00	361	2.23
**GACIN1**	5.52	11.00	381	2.10
**GACIN2**	10.09	7.38	370	1.75
**GACIN3**	5.58	13.32	382	2.33
**BZTidr4**	2.31	3.66	373	1.73
**PBSA**	5.00	1.01	319	0.16

**Table 5:** Antiproliferative activity of for hydrazine and acyl-hydrazone derivatives towards HaCaT (immortalized human keratinocyte cell line), Colo-38 (human melanoma cells), and A375 (human melanoma cells). All data are the mean of at least three independent experiments. (-, not tested).

Compound	HaCat (IC_50_, µM)	Colo38 (IC_50_, µM)	SI	A375 (IC_50_, µM)	SI
**GAHYDR1**	>100	89.60 ± 5.50	>1.12	80.65 ± 13.08	>1.24
**GAHYDR2**	38.59 ± 0.71	>100	<1	>100	<1
**GAHYDR3**	71.13 ± 4.67	>100	<1	8.75 ± 1.48	8.12
**BZTidr10**	89.27 ± 33.72	54.61 ± 12.21	1.63	-	-
**GACIN1**	80.64 ± 5.40	79.93 ± 12.71	1.01	>100	<1
**GACIN2**	37.90 ± 4.27	97.35 ± 0.78	<1	>100	<1
**GACIN3**	65.70 ± 5.64	>100	<1	>100	<1
**BZTidr4**	>100	92.85 ± 1.62	>1.08	-	-
**Cisplatin**	4.75 ± 0.35	-	-	<1.00	-
**[Cmp 13]**	-	0.62 ± 0.04	-	-	-

## Data Availability

The original contributions presented in this study are included in the article/Supplementary Materials. Further inquiries can be directed to the corresponding author.

## Supplementary Materials

The following supporting information can be downloaded at: https://www.mdpi.com/article/10.3390/molecules31132245/s1. Copies of ^1^H and ^13^C NMR spectra of new compounds (Figures S1–S12). Figure S1. ^1^H-NMR spectrum of compound **GAHYDR1.** Figure S2. ^13^C-NMR spectrum of compound **GAHYDR1.** Figure S3. ^1^H-NMR spectrum of compound **GAHYDR2.** Figure S4. ^13^C-NMR spectrum of compound **GAHYDR2.** Figure S5. ^1^H-NMR spectrum of compound **GAHYDR3.** Figure S6. ^13^C-NMR spectrum of compound **GAHYDR3.** Figure S7. ^1^H-NMR spectrum of compound **GACIN1.** Figure S8. ^13^C-NMR spectrum of compound **GACIN1.** Figure S9. ^1^H-NMR spectrum of compound **GACIN2.** Figure S10. ^13^C-NMR spectrum of compound **GACIN2.** Figure S11. ^1^H-NMR spectrum of compound **GACIN3.** Figure S12. ^13^C-NMR spectrum of compound **GACIN3**. Table S1: Preliminary in silico drug-likeness assessment.
