# Supplementary material for: New Hybrid Benzothiazole Derivatives from Gallic and Syringic Acid as a Potential Multifunctional Skin Disease
Source: Molecules. 2026 Jun 25;31(13):2245. doi: 10.3390/molecules31132245 (PMC13362733; doi:10.3390/molecules31132245)
Supplement: Supplementary file 1 [file molecules-31-02245-s001.zip › molecules-4388697-supplementary.pdf]

New Hybrid Benzothiazole Derivatives from Gallic and Syringic Acid as a Potential Multifunctional Skin Disease

Leonardo Montani [1], Chiara Tupini [2], Filippo Marchetti [1], Alessandra Rizzo [3], Silvia Vertuani [1], Stefano Manfredini [1], Ilaria Lampronti [2] and Anna Baldisserotto [1,*]

[1] Department of Life Sciences and Biotechnology, Section of Medicine and Health Products, University of Ferrara, via Fossato di Mortara 19, 44121 Ferrara, Italy; leonardo.montani@unife.it (L.M.); filippo.marchetti@unife.it (F.M.); vrs@unife.it (S.V.); mv9@unife.it (S.M.)

[2] Department of Life Sciences and Biotechnology, Section of Biochemistry and Molecular Biology, University of Ferrara, via Fossato di Mortara 74, 44121 Ferrara, Italy; chiara.tupini@unife.it (C.T.); ilaria.lampronti@unife.it (I.L.)

[3] Department of Chemical, Pharmaceutical and Agricultural Sciences, University of Ferrara, via Luigi Borsari 46, 44121 Ferrara, Italy; alessandra.rizzo@unife.it

* Correspondence: bldnna@unife.it; Tel.: +39-0532-455258

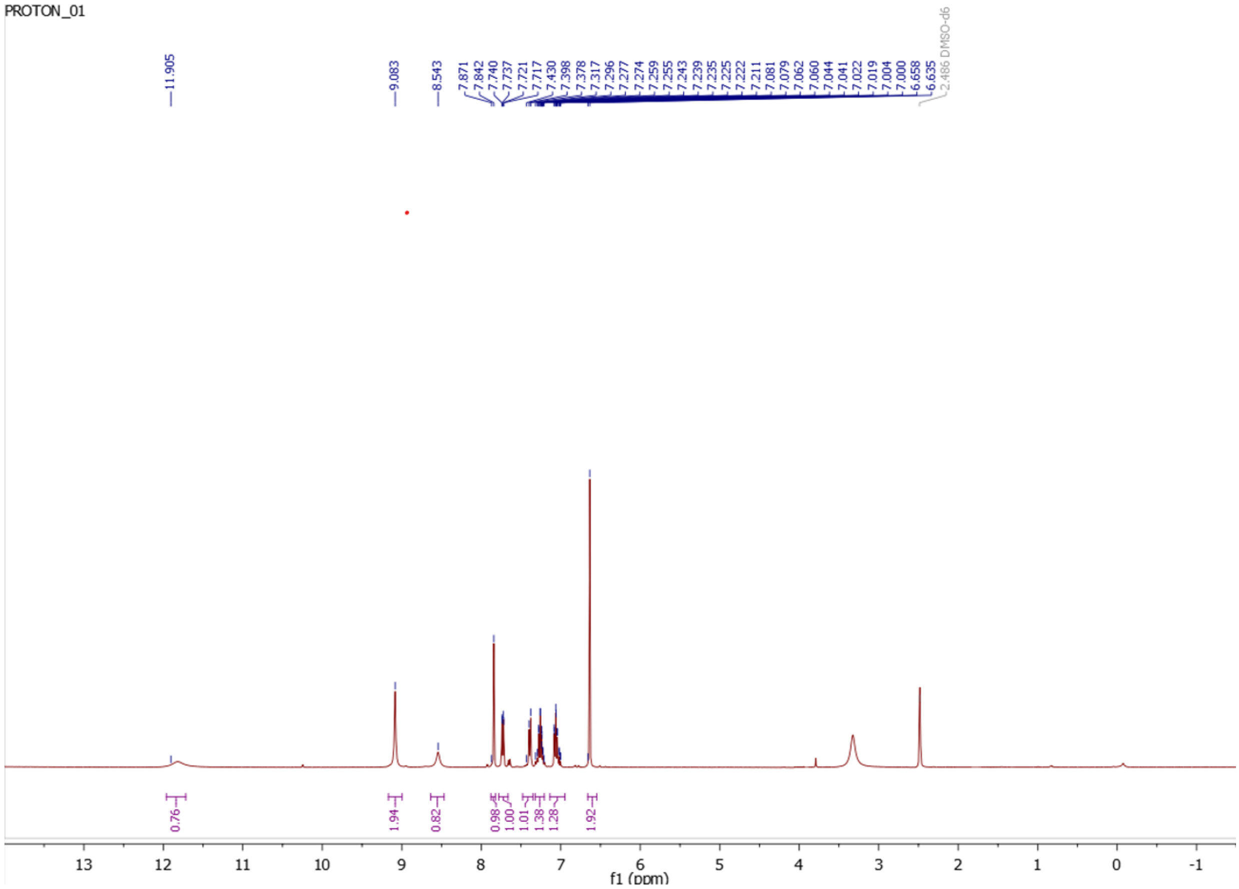

Figure S1. ^{1}H-NMR spectrum of compound **GAHYDR1.**

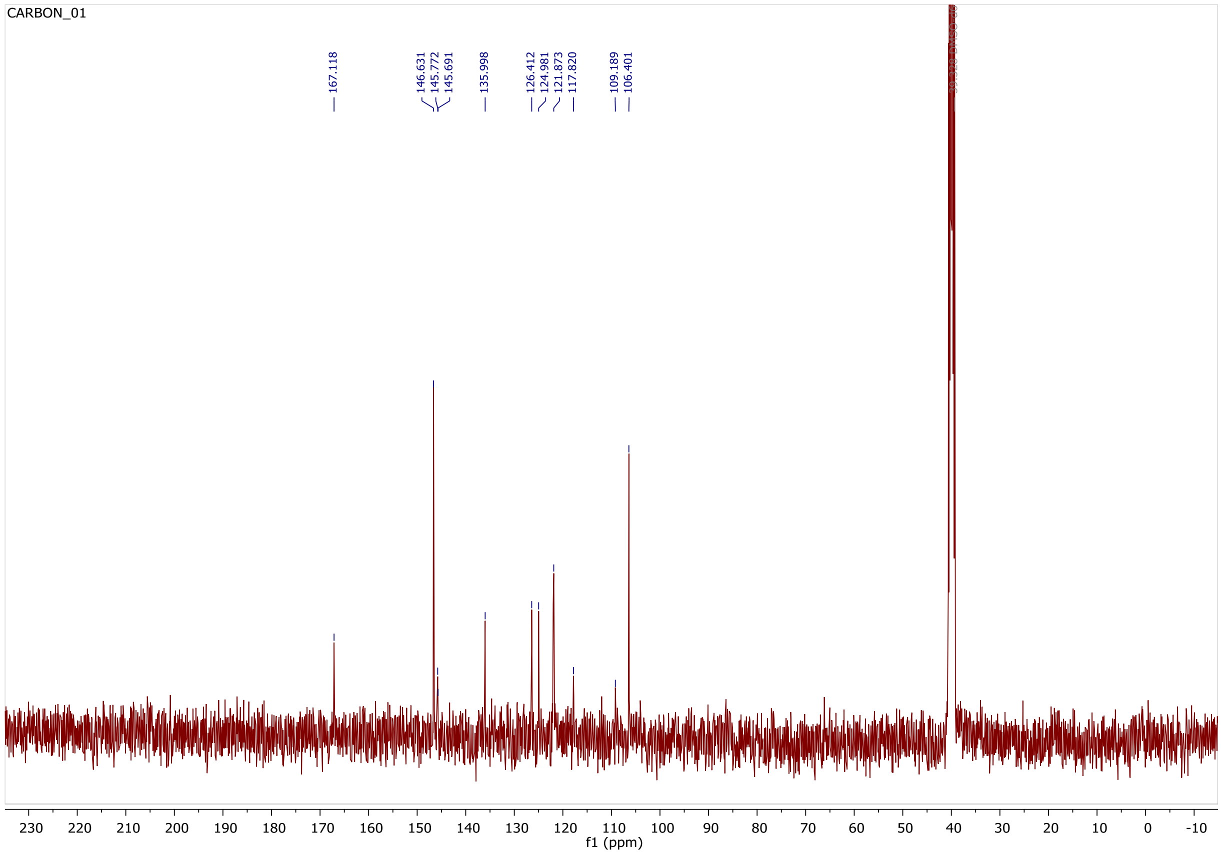

Figure S2. ^{13}C-NMR spectrum of compound **GAHYDR1.**

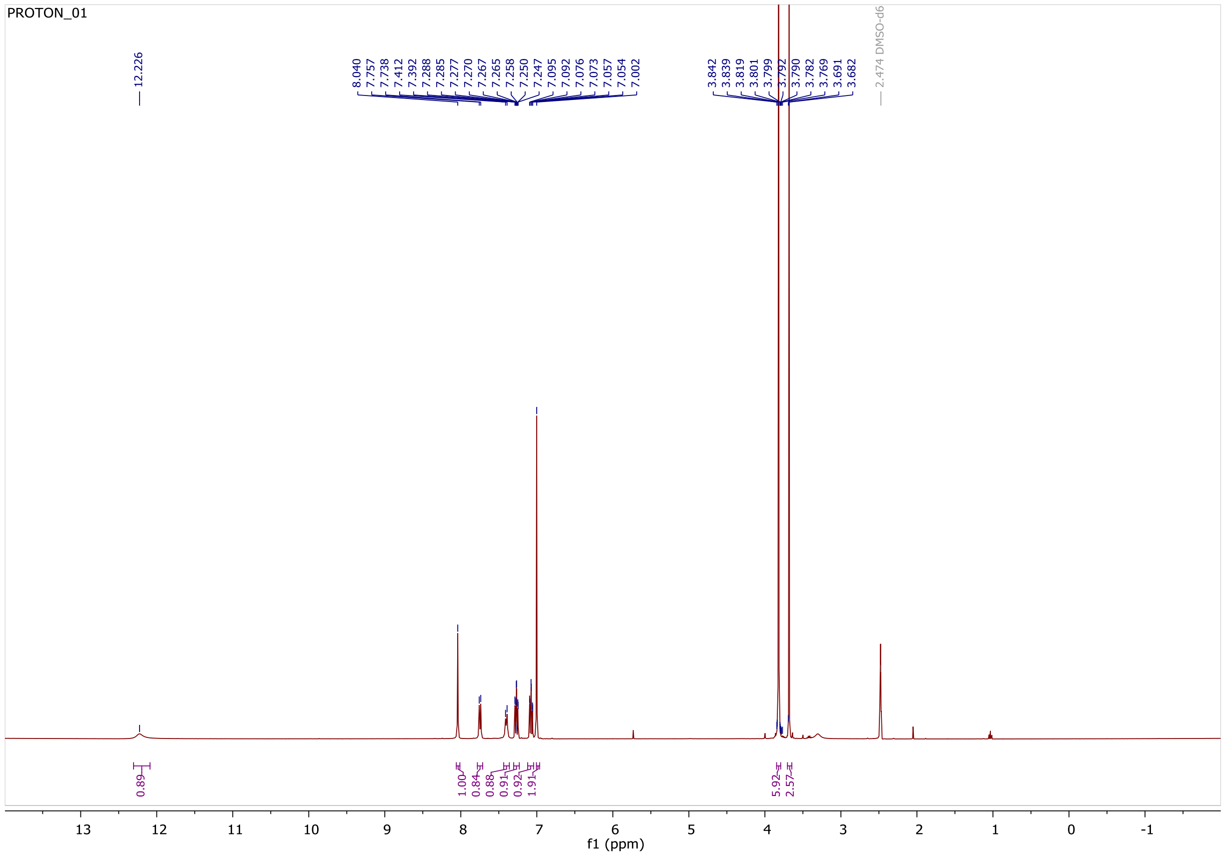

Figure S3. ^{1}H-NMR spectrum of compound **GAHYDR2.**

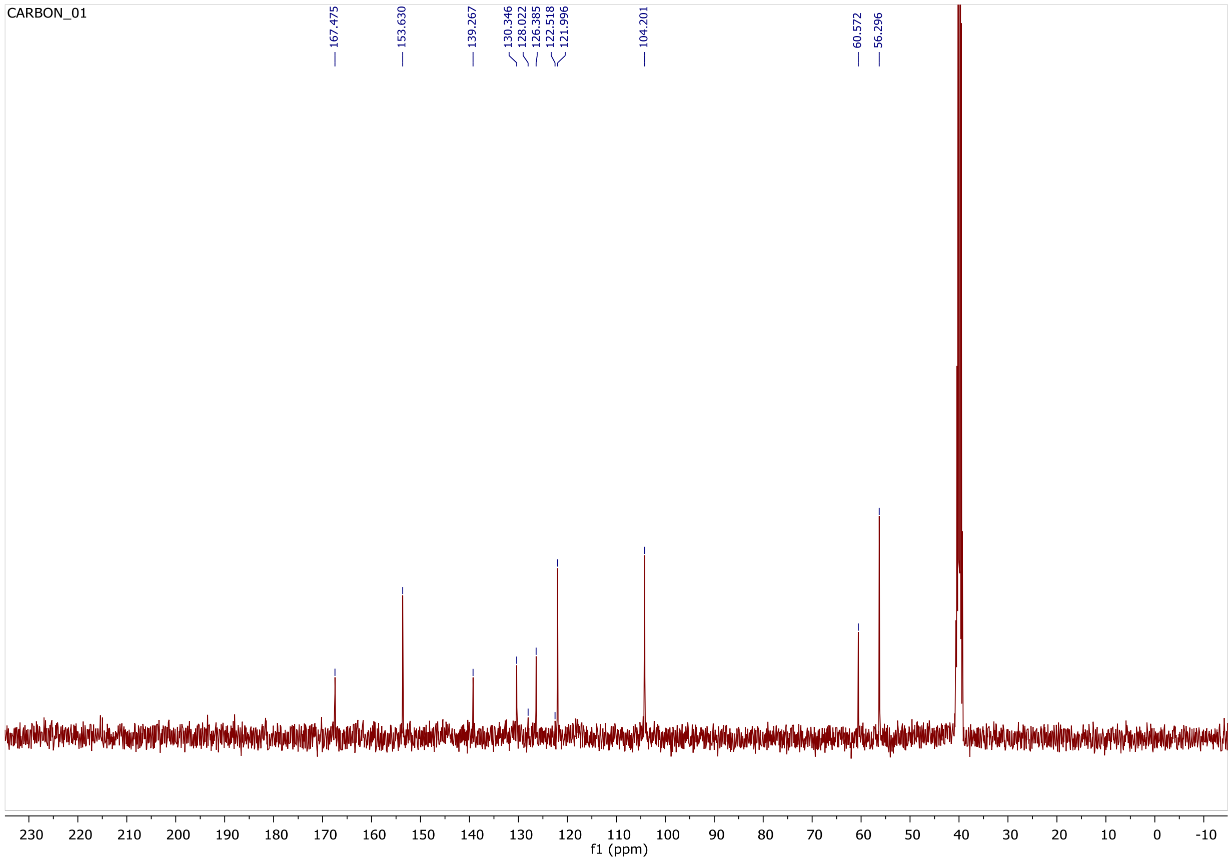

Figure S4. ^{13}C-NMR spectrum of compound **GAHYDR2.**

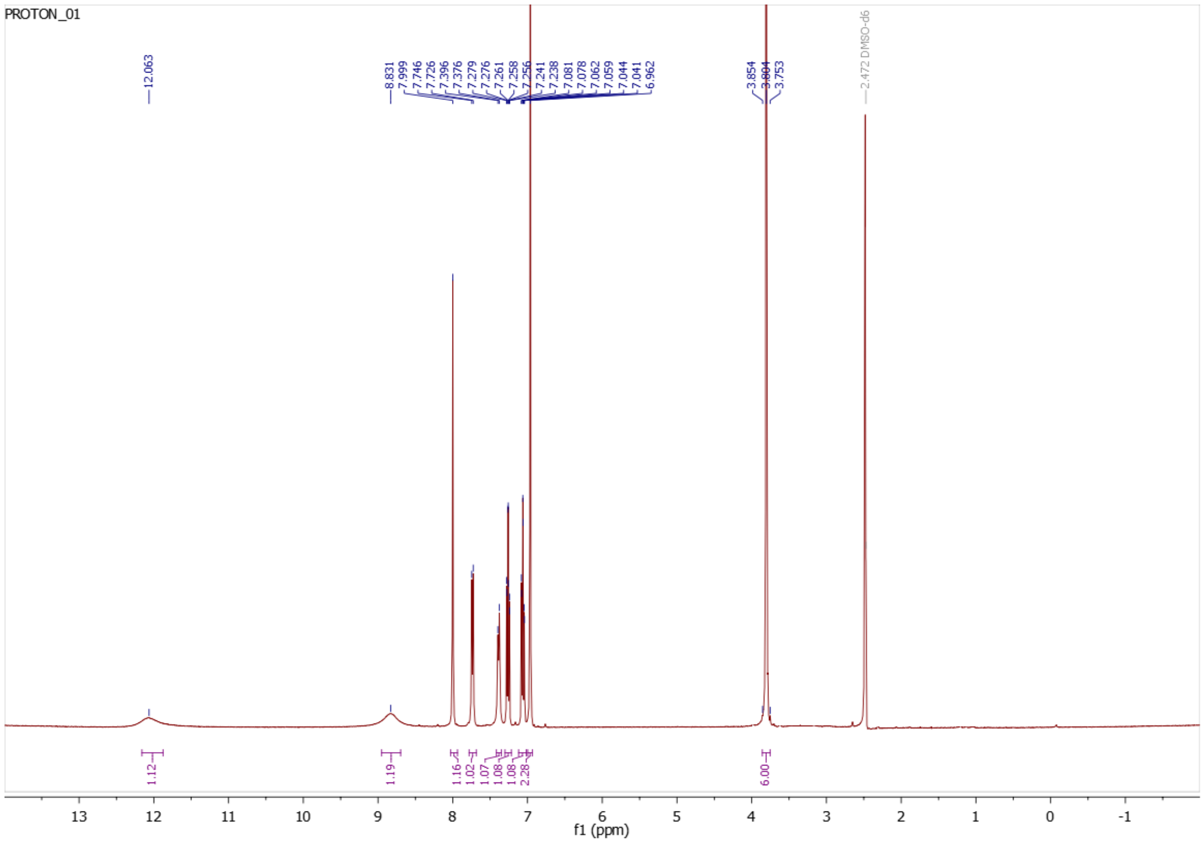

Figure S5. ^{1}H-NMR spectrum of compound **GAHYDR3.**

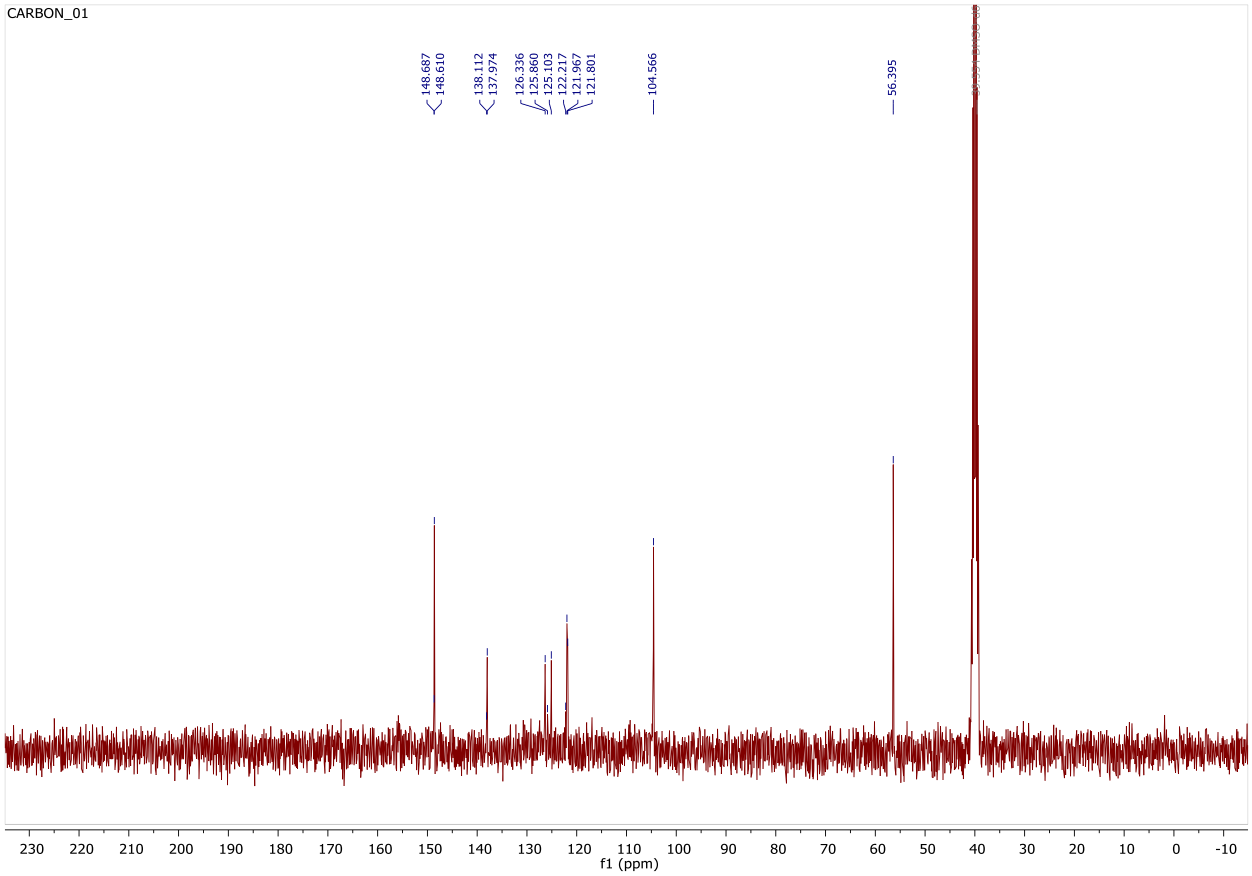

Figure S6. ^{13}C-NMR spectrum of compound **GAHYDR3.**

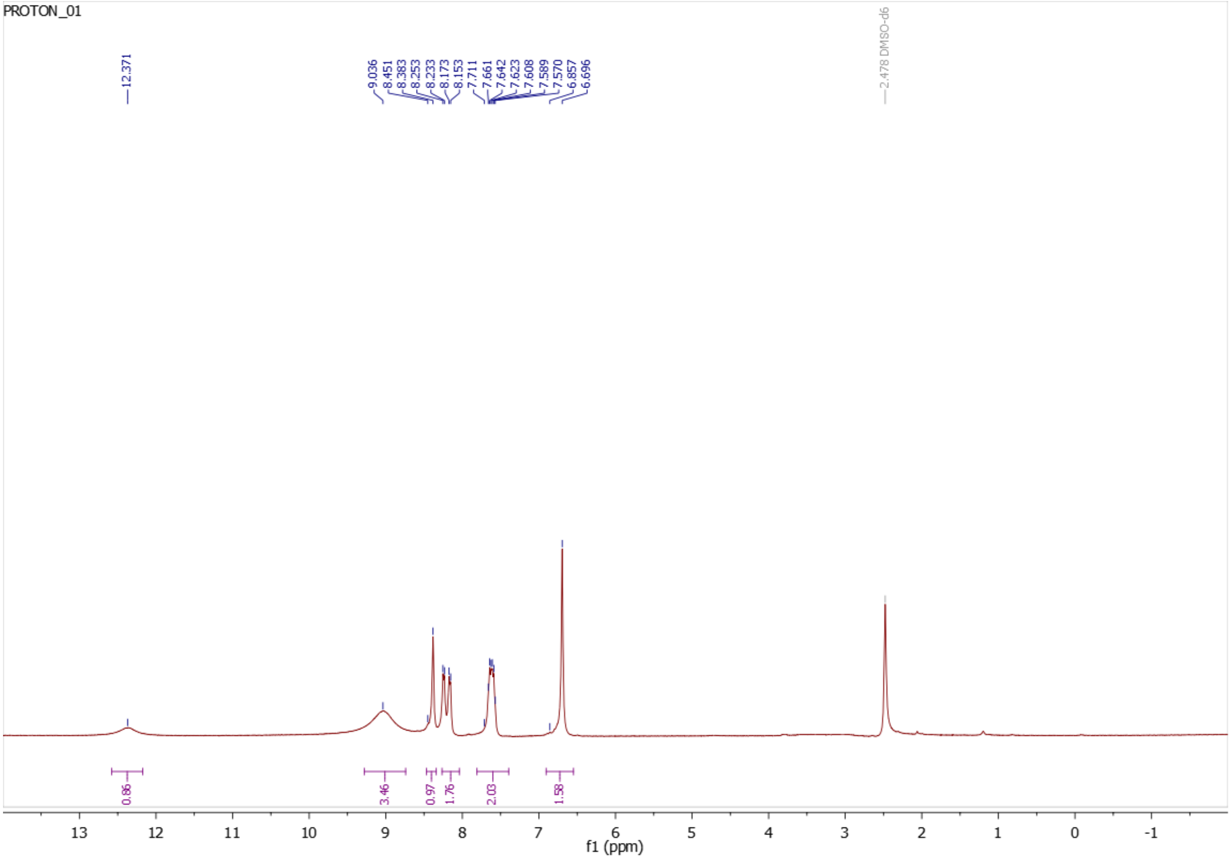

Figure S7. ^{1}H-NMR spectrum of compound **GACIN1.**

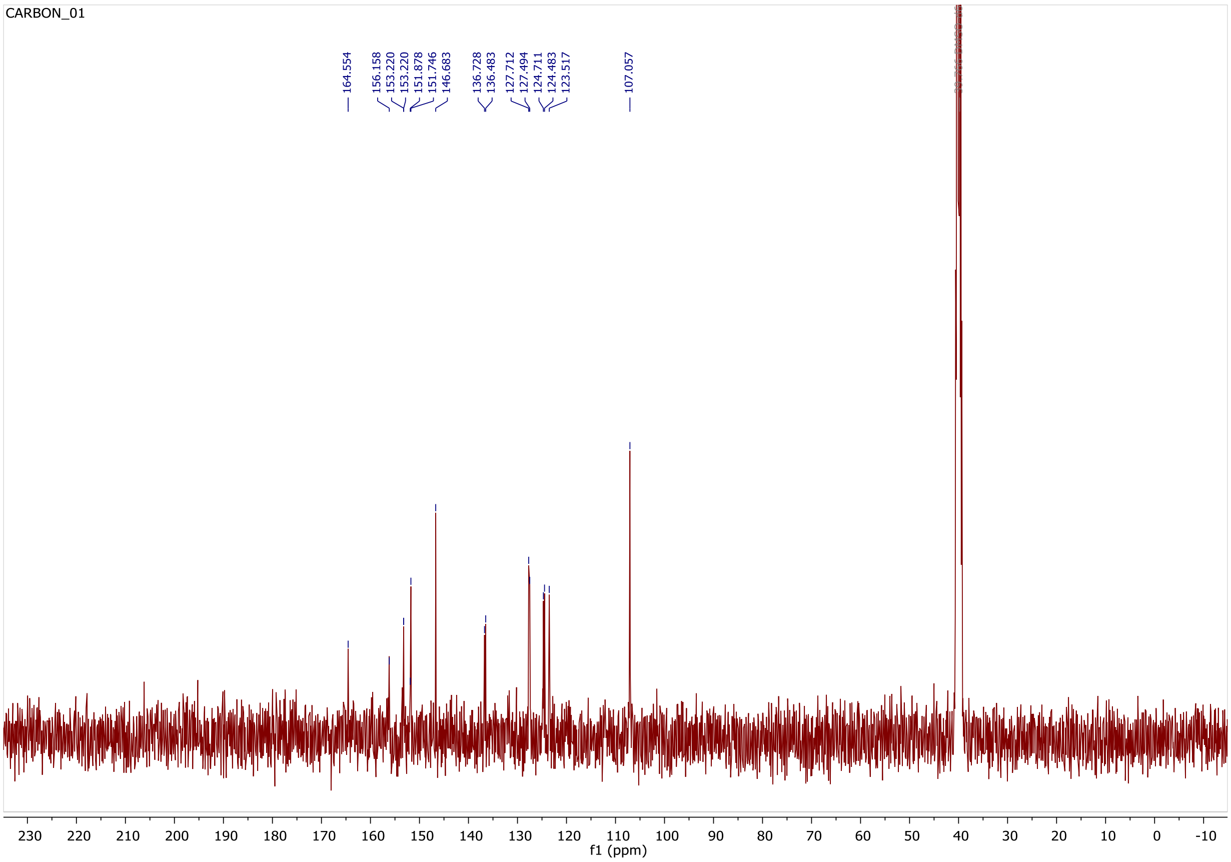

Figure S8. ^{13}C-NMR spectrum of compound **GACIN1.**

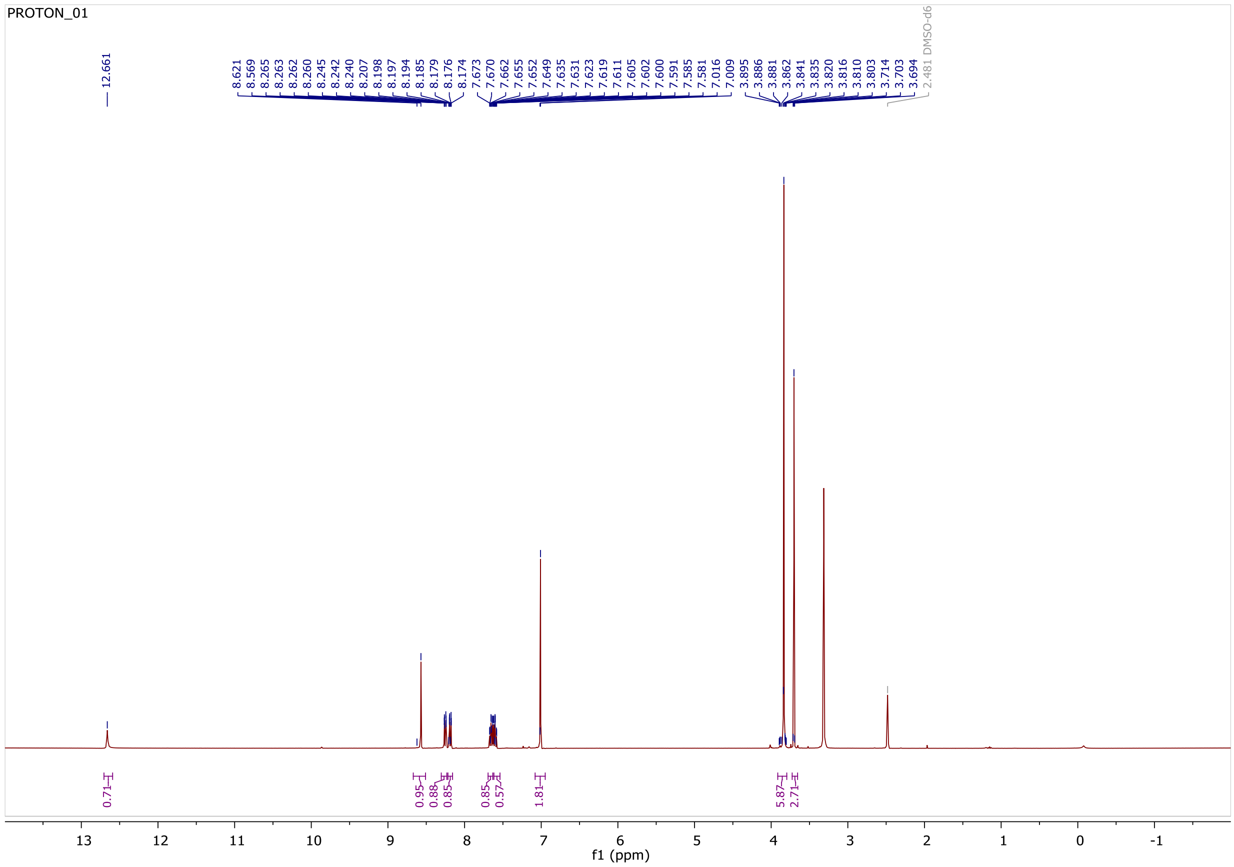

Figure S9. ^{1}H-NMR spectrum of compound **GACIN2.**

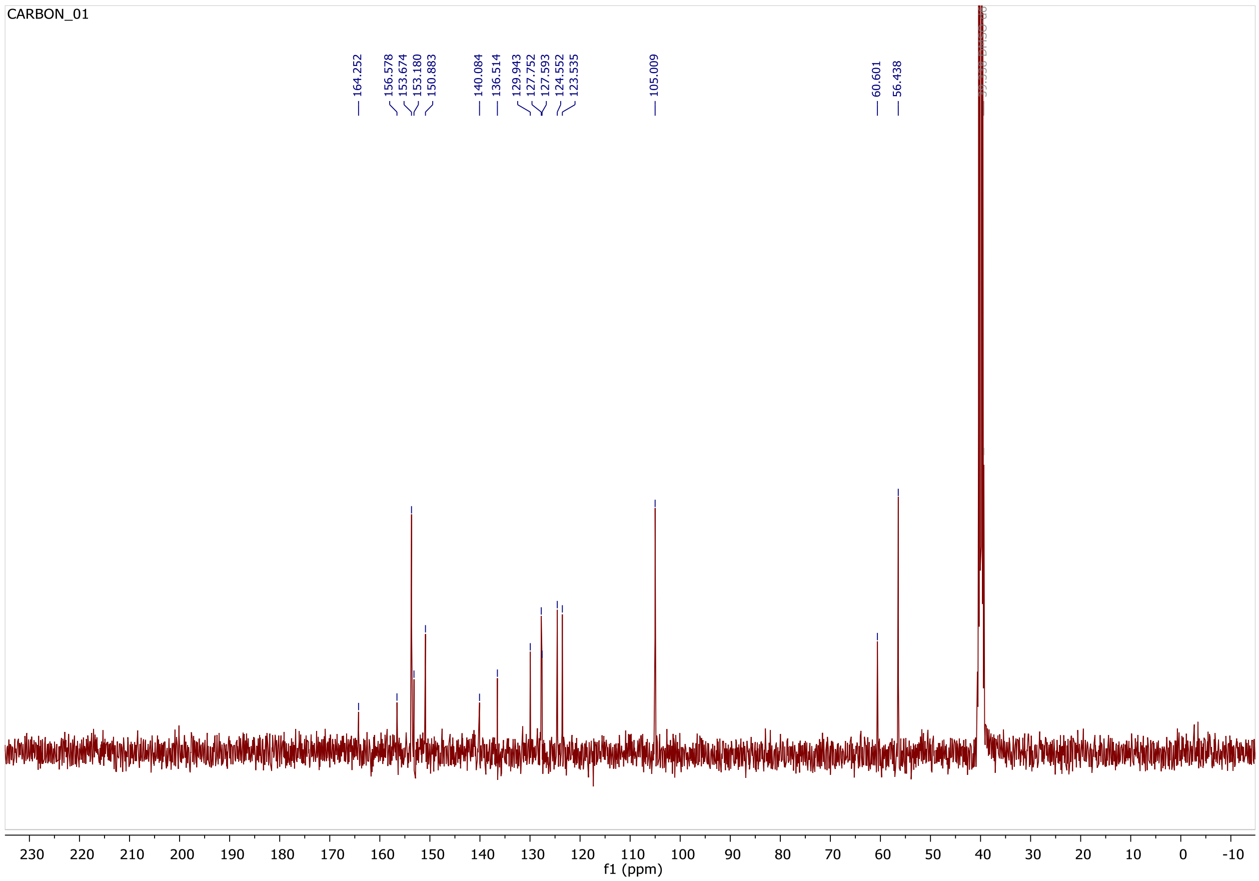

Figure S10. ^{13}C-NMR spectrum of compound **GACIN2.**

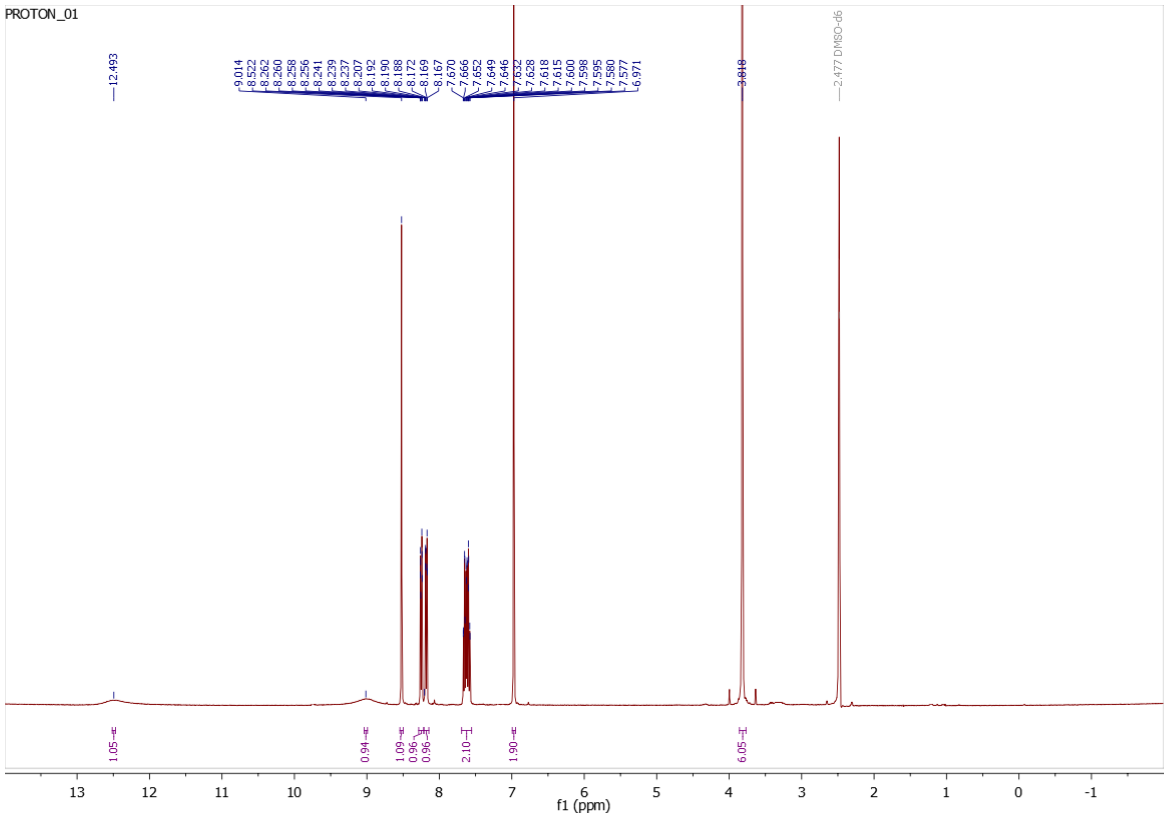

Figure S11. ^{1}H-NMR spectrum of compound **GACIN3.**

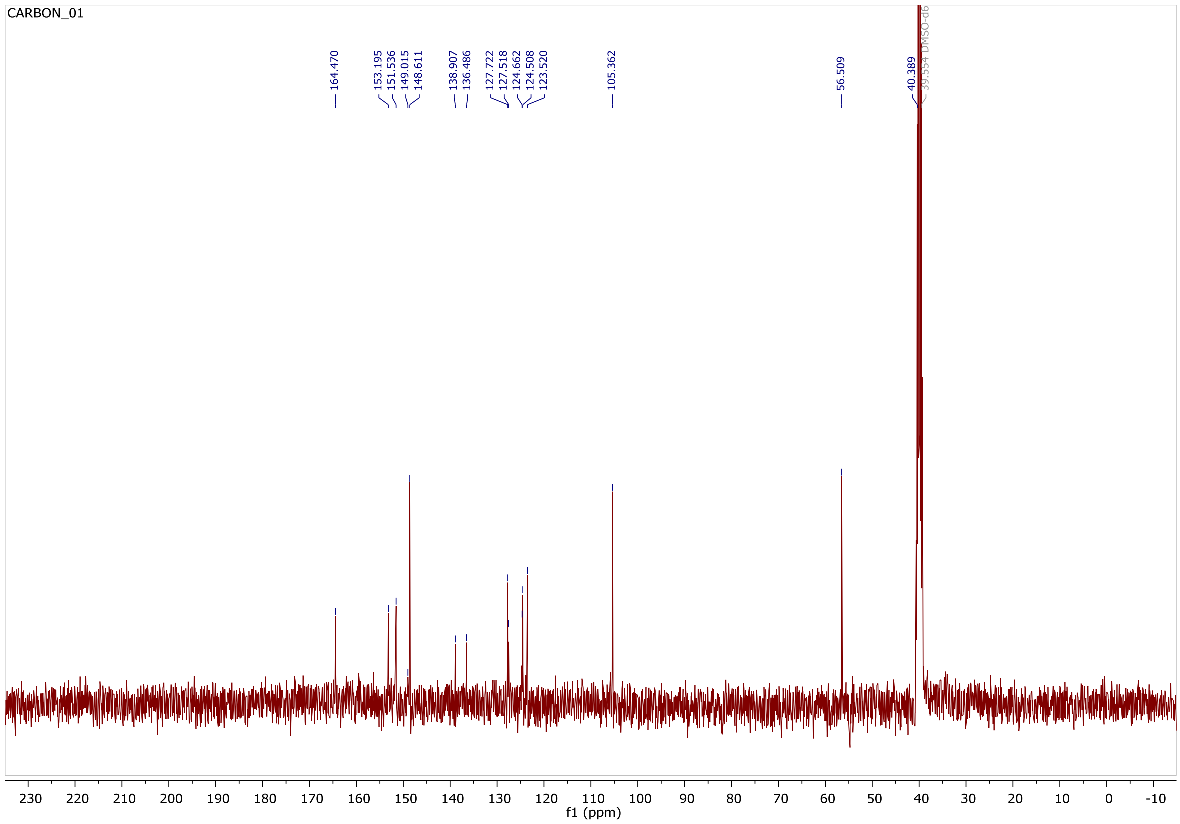

Figure S12. ^{13}C-NMR spectrum of compound **GACIN3.**

**Molecular weight**	329.37 g/mol
**Consensus LogP**	3.18
**GI absorption**	high
**Lipinski**	YES (0 violations)
**Veber**	YES
**Egan**	YES
**Ghose**	YES
**Bioavailability score**	0.55
**Synthetic accessibility**	3.03

Table S1. Preliminary in silico drug-likeness assessment.
